# Case Report: A Case of Renal Cell Carcinoma Unclassified With Medullary Phenotype Exhibiting a Favorable Response to Combined Immune Checkpoint Blockade

**DOI:** 10.3389/fimmu.2022.934991

**Published:** 2022-07-05

**Authors:** Masashi Takeda, Soki Kashima, Yasushi Fuchigami, Takayuki Yoshino, Tatsuki R. Kataoka, Toshinari Yamasaki, Hiroshi Kagamu, Takashi Kobayashi, Shusuke Akamatsu

**Affiliations:** ^1^ Department of Urology, Kyoto University Graduate School of Medicine, Kyoto, Japan; ^2^ Department of Urology, Akita University Graduate School of Medicine, Akita, Japan; ^3^ Department of Urology, Kurashiki Central Hospital, Okayama, Japan; ^4^ Department of Urology, Faculty of Medicine, University of Tsukuba, Ibaraki, Japan; ^5^ Department of Diagnostic Pathology, Kyoto University Graduate School of Medicine, Kyoto, Japan; ^6^ Department of Pathology, Iwate Medical University, Iwate, Japan; ^7^ Department of Urology, Kobe City Medical Center General Hospital, Hyogo, Japan; ^8^ Division of Respiratory Medicine, Saitama Medical University International Medical Center, Saitama, Japan

**Keywords:** Renal cell Caecinoma, immune checkpoint inhibitors, RCCU-MP, case report, ipilimumab, Nivolumab

## Abstract

Renal cell carcinoma unclassified with medullary phenotype (RCCU-MP) is an extremely rare variant of kidney cancer with poor prognosis. Recently, immune checkpoint inhibitors (ICIs) have been the mainstay of treatment for advanced clear cell renal cell carcinoma (RCC). However, the efficacy of ICI in the treatment of RCCU-MP remains unclear. Here, we report about a 63-year-old Japanese man who was referred to our hospital with a diagnosis of RCC of the left kidney with multiple–lymph node involvement (cT3aN1M1). The patient underwent nephrectomy with lymph node biopsy, which was histopathologically diagnosed as RCCU-MP. Thereafter, he received combined immune checkpoint blockade with nivolumab and ipilimumab. After induction therapy, follow-up computed tomography revealed shrinkage of the metastatic lymph nodes. Moreover, the patient was relieved of his subjective symptoms and his performance status improved. However, after 15 months, maintenance ICI therapy was discontinued because of disease progression, and the patient died 28 months after diagnosis. Longitudinal analysis of peripheral blood mononuclear cells revealed increased stem cell memory and central memory CD8^+^ T-cell subsets during response to therapy and enhanced expression of exhaustion markers on CD8^+^ T cells upon treatment resistance. Combined immune checkpoint blockade could be effective in the treatment of metastatic RCCU-MP.

## Introduction

Renal medullary carcinoma (RMC) is a rare and aggressive type of kidney cancer, typically associated with sickle cell trait. Clinicopathologically, RMC is characterized by the loss of SWI/SNF-related matrix–associated actin–dependent regulator of chromatin subfamily B member (SMARCB1)/integrase interactor 1 (INI1) protein (1). RMC without sickle cell trait, classified as renal cell carcinoma unclassified with medullary phenotype (RCCU-MP), is an extremely rare variant of kidney cancer. A small number of case reports suggest that RCCU-MP is associated with poor prognosis due to an unfavorable response to molecular-targeted therapies and chemotherapy (2, 3). In contrast, the effect of immune checkpoint inhibitors (ICIs) on RCCU-MP remains unknown. Here, we report a case of RCCU-MP showing a favorable response to combined immune checkpoint blockade therapy with PD-1 inhibitor nivolumab and CTLA-4 inhibitor ipilimumab. To the best of our knowledge, this is the first report to describe a case of RCCU-MP treated with ICI.

In addition to case presentation, we report the results of mass cytometry and multicolor flow cytometry analysis of peripheral blood mononuclear cells (PBMCs) collected from the patient at three different time points during ICI treatment.

## Case Report

**Figure 1:**
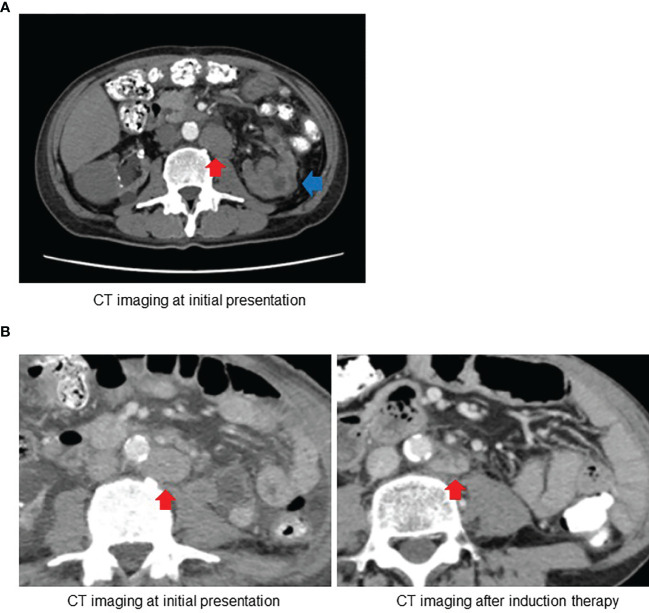
**(A)** Computed tomography (CT) at initial presentation revealed the left kidney tumor (blue arrow) with para-aortic lymph node involvement (red arrow). **(B)** Follow-up CT after induction therapy with ipilimumab and nivolumab showed substantial decrease in size of para-aortic lymph node.

A 63-year-old Japanese man presented to the referring hospital with a 1-month history of fever, back pain, and macroscopic hematuria. He had been undergoing hemodialysis for past 15 years for chronic kidney disease of unknown origin. Computed tomography (CT) showed a poorly enhanced tumor within the upper pole of the left kidney measuring 1.4 cm × 3.6 cm × 2.2 cm with multiple–lymph node involvement including para-aortic, retrocrural, and supraclavicular lymph nodes (**Figure 1**). Metastasis to distant organ was not detected. A clinical diagnosis of RCC (cT3aN1M1) was made, and the patient was referred to our hospital for treatment.

As the patient was symptomatic from the local disease and was already on hemodialysis, radical nephrectomy and para-aortic lymph node biopsy were performed. The pathological characteristics of this case has already been published (4). Histologically, the tumor consisted of rhabdoid cells, with notable lymphocyte infiltration and large areas of necrosis. Immunohistochemically, the tumor was negative for SMARCB1, and PD-L1 expression was observed in 20% of cells (**Supplementary Data 1**). All sampled lymph nodes were positive for malignancy. Because the patient had no evidence of sickle cell trait or sickle cell disease, he was diagnosed as having RCCU-MP (pT3a, pN1).

One month after nephrectomy, the baseline CT scan showed para-aortic lymph node measuring 40 mm × 30 mm, retrocrural lymph node measuring 27 mm × 19 mm, and left supraclavicular lymph node measuring 23 mm × 21 mm. We administered a combined immune checkpoint blockade therapy with nivolumab at a dose of 240 mg and ipilimumab at a dose of 1 mg/kg every 3 weeks as induction therapy for the management of residual lesions. After four cycles of induction therapy without grade 3 or 4 immune-related adverse events, follow-up CT revealed partial responses with a substantial decrease in the size of the affected lymph nodes (**Supplementary Data 2**). The patient became afebrile, and his performance status evidently improved by the induction therapy. In addition, there was a significant reduction in serum C-reactive protein (CRP) levels.

Taking the results into consideration, we considered induction therapy to be effective, and the patient continued maintenance therapy with nivolumab at a dose of 240 mg every 2 weeks. After 15 months of maintenance nivolumab therapy without disease progression, the patient presented with left lower limb edema because of left common and external iliac lymphadenopathy. Palliative radiation therapy at a dose of 50 Gy relieved the patient’s symptoms.

However, after 1 month, his blood test results showed abnormal liver function, and CT revealed obstructive jaundice requiring biliary drainage. We considered this situation as disease progression, discontinued nivolumab, and switched the therapy to chemotherapy consisting of carboplatin and paclitaxel. Chemotherapy was discontinued after two cycles due to declined performance status and side effects of myalgia. The patient received best supportive care and died 2 months later due to disease progression. The total time span between diagnosis and death was 28 months.

To explore the longitudinal change in the profile of circulating T lymphocytes during ICI treatment, we performed mass cytometry and multicolor flow cytometry analysis of PBMCs isolated from the patient’s blood samples, which were obtained at three different time points: beginning, end of induction therapy, and when the disease had progressed. This analysis was conducted according to the protocol described by Kagamu et al. (5).

**Figure 2:**
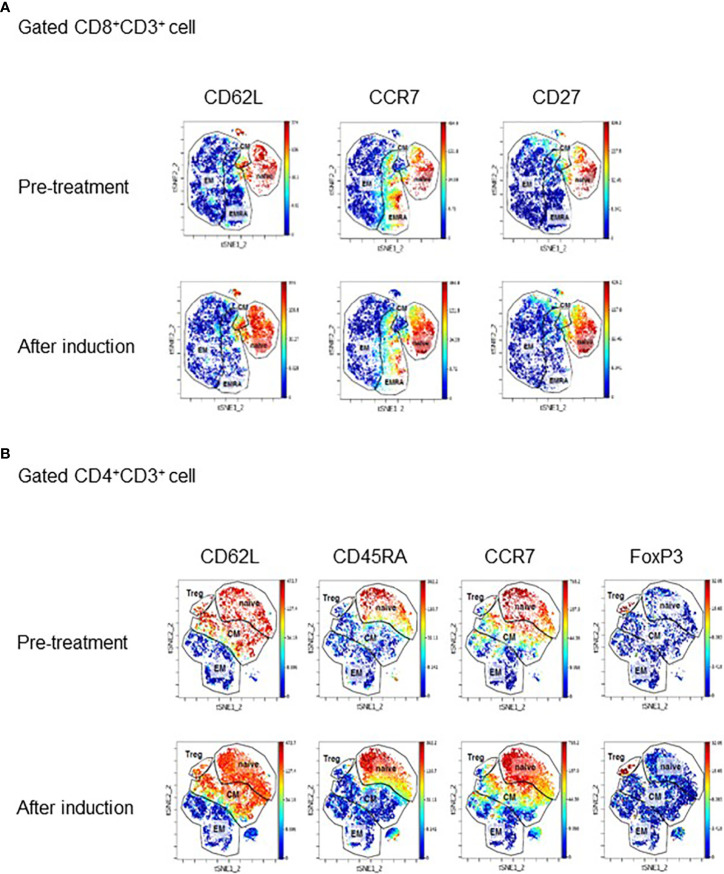
**(A)** Mass cytometry data showing changes in expression of CD62L, CCR7, and CD27 in CD8^+^ and CD4^+^ T cells. The data are visualized using the t-distributed stochastic neighbor embedding (t-SNE) technique. PBMC samples were collected before ICI treatment and after induction. **(B)** tSNE plot displaying subpopulations of CD8^+^ cells **(A)** and CD4^+^ cells **(B)** determined by mass cytometry before treatment and after induction therapy when tumor is responding to therapy. Major subpopulations are surrounded by lines. EM, effector memory; CM, central memory; EMRA, effector memory cells re-expressing CD45RA; Treg, regulatory T cell.

PBMC samples obtained before treatment and at the completion of induction therapy, when treatment response was evident, were subjected to mass cytometry. Mass cytometry data visualized using t-distributed stochastic neighbor embedding (t-SNE) technique revealed an apparent increase in stem cell memory and central memory CD8^+^ T cells expressing CD62L, CCR7, and CD27 (**Figure 2A**) . This finding was supported by multicolor flow cytometry analysis showing an increase in the proportion of central memory (CD45RA^−^CCR7^+^) and naïve (CD45RA^+^CCR7^+^) CD8^+^ T cells after induction therapy, whereas the proportion of effector (CD45RA^+^CCR7^−^) and effector memory (CD45RA^−^CCR7^−^) CD8^+^ T cells decreased (**Supplementary Data 3A**). A similar increase in the stem-like population was also observed in CD4^+^ T cells (**Figure 2B**). Upon treatment resistance, the proportion of effector T cells increased significantly and the proportion of central memory and naïve CD8^+^ T cells decreased (**Supplementary Data 3A**). Multicolor flow cytometry analysis also revealed that the population expressing exhaustion markers such as PD-1^+^, LAG-3^+^, and CTLA-4^+^ consistently increased upon treatment resistance across all CD8^+^ T cells and Th1 CD4^+^ cells (**Supplementary Data 3B**). In addition, the fraction of CD62L^low^CD4^+^ T cells among total CD4^+^ T cells before treatment, a parameter previously reported to be associated with response to ICI in non–small cell lung cancer, was 39.6%, classifying the present case to the responder group (5).

## Discussion

RCCU-MP is an extremely rare variant of renal cancer with poor survival. Very few cases have been published to date (**Supplementary Data 4**). Sirohi et al. described five cases of RCCU-MP. In four cases, patients died of the disease with survival time ranging from 3 to 27 months (3). Because of the paucity of evidence, we decided to treat our case based on the findings of RMC, which shares pathological features with RCCU-MP.

RMC is also a rare, aggressive type of renal cancer, strongly associated with sickle cell trait or sickle cell disease. Most patients present with a metastatic disease. RMC is considered to originate from the collecting duct, and chemotherapy is commonly used for its management. However, several reports have demonstrated poor outcomes. Ezekian et al. reported 159 cases of RMC, wherein majority of the patients underwent surgery (60%) and chemotherapy (65%), with a median survival of 7.7 months (6).

Recently, a few case reports describing RMC with a durable response to ICI have been published. Beckermann et al. reported a case of recurring RMC with lymph node metastasis, which showed complete response to PD-1 blockade, lasting for more than 9 months (7). Sodji et al. described two cases of RMC treated with nivolumab (8). In this report, one patient showed durable response with nivolumab for more than 15 months, whereas the other case showed disease progression in 3 months. In our case, the patient showed durable response to ICI for more than 15 months. It is intriguing that immunotherapy is also reported to be effective in soft tissue sarcoma with SMARCB1 loss (9, 10). Although the mechanism is unknown, it is possible that immunotherapy could be effective for SMARCB1-deficient tumors regardless of the tumor type. Future accumulation of data is awaited.

To the best of our knowledge, this is the first case report describing RCCU-MP treated with ICI. Two studies reported RCCU-MP cases treated with molecular-targeted therapy. Lai et al. reported a case of metastatic RCCU-MP treated with everolimus and bevacizumab, with unknown outcomes (2). Colombo et al. also reported a case of metastatic RCCU-MP treated with sunitinib and sorafenib; however, the patient died within 10 months of follow-up (11). Combination therapy with tyrosine kinase inhibitors (TKIs) predominantly targeting vascular endothelial growth factor and ICI is the standard treatment for ccRCC. Whether TKIs have additive or synergistic effect to ICI in RCCU-MP characterized by SMARCB1 loss remains unknown. Further accretion of RCCU-MP cases treated with a variety of combination therapies including ICI is necessary to determine the optimal treatment for this extremely rare variant of renal cancer.

To examine the change in the profile of circulating T lymphocytes during ICI treatment, we performed mass cytometric analysis and multicolor flow cytometry analysis of PBMC. The results showed a shift of CD8^+^ T cell to naïve and central memory subpopulation, which are less exhausted and reported to possess strong anti-tumor activity during response to treatment (12). This phenomenon could be attributable to CTLA-4 blockade that activates and recruits CD8^+^ T cell subpopulation distinct from those activated by anti–PD-1 monotherapy through activation of CD4^+^ T cells, especially Th1-like T cells (13, 14). Mass cytometry and multicolor flow cytometry also showed decreased expression of PD-1 during response to ICI and exhaustion of T cells upon treatment resistance, as evidenced by an increased population expressing PD-1, LAG-3, and CTLA-4. In addition, CD62L^low^CD4^+^ T cells, which are reported to be predictive of response to ICI in non–small cell lung cancer, were also enriched in the peripheral blood, favoring response to ICI. Taken together, our data clearly show a dynamic shift in peripheral blood markers following ICI, suggesting the potential for development of peripheral blood markers to predict response to ICI.

The patients with 1% or greater PD-L1 expression have been demonstrated to receive significant clinical benefits from ipilimumab plus nivolumab compared with nivolumab alone. In contrast, those with less than 1% PD-L1 expression showed comparable outcomes to both treatment (15, 16). Thus, selection of ipilimumab plus nivolumab in the present case was reasonable. In the present case, LAG-3–expressing T cells were increased at the time of disease progression. This may, in turn, imply that anti–LAG-3 therapy could have been effective in our case. In patients with advanced malignant melanoma, anti–LAG-3 antibody relatlimab has shown superior outcome in combination with nivolumab compared with nivolumab alone (17). Further research is required to explore novel ICI combination based on tumor microenvironment and, possibly, on peripheral blood markers as well.

The limitation of this study was the absence of sequencing data of tissue-infiltrating lymphocytes (TILs), which would allow analysis of T-cell immunity in the tumor microenvironment, as well as the interaction between T cells in the tumor microenvironment and circulation. The TIL analysis could not be done due to the lack of sufficient viable cells in nephrectomy specimens for sequencing. It has been reported that central memory CD8^+^ T cells in TIL increase in those responding to ICI (18). Comprehensive analysis involving peripheral T cells and TIL is required to elucidate integrated T-cell immunity in response to ICI.

We presented the first report describing RCCU-MP treated with ICI therapy. The patient showed durable response to a combination of ipilimumab and nivolumab. Further research involving larger cohort is needed to develop effective combined ICI treatment for RCCU-MP.

## Data Availability Statement

The original contributions presented in the study are included in the article/**Supplementary Material**. Further inquiries can be directed to the corresponding authors.

## Ethics Statement

The studies involving human participants were reviewed and approved by Kyoto University’s Institutional Board (approval number G52). The patients/participants provided their written informed consent to participate in this study. Written informed consent was obtained from the individual(s) for the publication of any potentially identifiable images or data included in this article.

## Author Contributions

MT collected data and drafted the manuscript. SK collected data and interpreted the results. TKa made the pathological diagnosis. YF and TYo cared for the study patient and collected clinical data. TYa edited the manuscript. HK performed the analysis and interpreted the results. TKo edited the manuscript. SA supervised the work, interpreted the results, and edited the manuscript. All authors contributed to the article and approved the submitted version.

## Conflict of Interest

The authors declare that the research was conducted in the absence of any commercial or financial relationships that could be construed as a potential conflict of interest.

## Publisher’s Note

All claims expressed in this article are solely those of the authors and do not necessarily represent those of their affiliated organizations, or those of the publisher, the editors and the reviewers. Any product that may be evaluated in this article, or claim that may be made by its manufacturer, is not guaranteed or endorsed by the publisher.

## Supplementary Material

Supplementary Data Sheet 1 Histological analysis of nephrectomy specimen. **(A)** H&E staining showing rhabdoid tumor cells (yellow arrows) with notable lymphocyte infiltration (red arrow) and large areas of necrosis (×200 magnification). **(B)** Immunohistochemically, the tumor is negative for SMARCB1 (×200 magnification). **(C)** PD-L1 expression was seen in 20% of the cells (×400 magnification).

Supplementary Data Sheet 2 Change in the size of metastatic lymph nodes during ICI therapy.

Supplementary Data Sheet 3
**(A)** Data of multicolor flow cytometry showed increase in the proportion of central memory T cell (CD45RA^-^CCR7^+^) and naïve T cell (CD45RA^+^CCR7^+^) during the course of ICI treatment, while the proportion of effector T cell (CD45RA^+^CCR7^-^) and effector memory T cell (CD45RA^-^CCR7^-^) decreased. The proportion of effector T cells re-elevated upon treatment resistance. **(B)** Change in the percentage of PD-1^+^, CTLA-4^+^, and LAG-3^+^ subpopulation in naïve, central memory, effector memory, and effector CD8^+^ T cells and Th1 cells during the course of ICI treatment. Tcm, central memory T cell; Tem, effector memory T cell; Teff, effector T cell.

Supplementary Data Sheet 4 Summary of previous reports of RCCU-MP. N/A, not available; DOD, died of the disease.

## Abbreviations

RCCU-MP, renal cell carcinoma unclassified medullary phenotype; RMC, renal medullary carcinoma; ICI, immune checkpoint inhibitor; PD-1, programmed cell death 1; CTLA-4, cytotoxic T-lymphocyte–associated protein 4; LAG-3, lymphocyte antigen gene 3; SMARCB1, SWI/SNF-related matrix–associated actin–dependent regulator of chromatin subfamily B member 1; INI 1, integrase interactor 1; PBMC, peripheral blood mononuclear cell; CRP, C-reactive protein; CT, computed tomography; CD, cluster differentiation; t-SNE, t-distributed stochastic neighbor embedding; TIL, tissue-infiltrating lymphocyte; LAG, lymphocyte activation gene.
