# Supplementary material for: Case Report: A Case of Renal Cell Carcinoma Unclassified With Medullary Phenotype Exhibiting a Favorable Response to Combined Immune Checkpoint Blockade
Source: Front Immunol. 2022 Jul 5;13:934991. doi: 10.3389/fimmu.2022.934991 (PMC9294239; doi:10.3389/fimmu.2022.934991)
Supplement: Supplementary Data Sheet 1 — Histological analysis of nephrectomy specimen. (A) H&E staining showing rhabdoid tumor cells (yellow arrows) with notable lymphocyte infiltration (red arrow) and large areas of necrosis (×200 magnification). (B) Immunohistochemically, the tumor is negative for SMARCB1 (×200 magnification). (C) PD-L1 expression was seen in 20% of the cells (×400 magnification). [file DataSheet_1.pdf]

Supplementary data 1

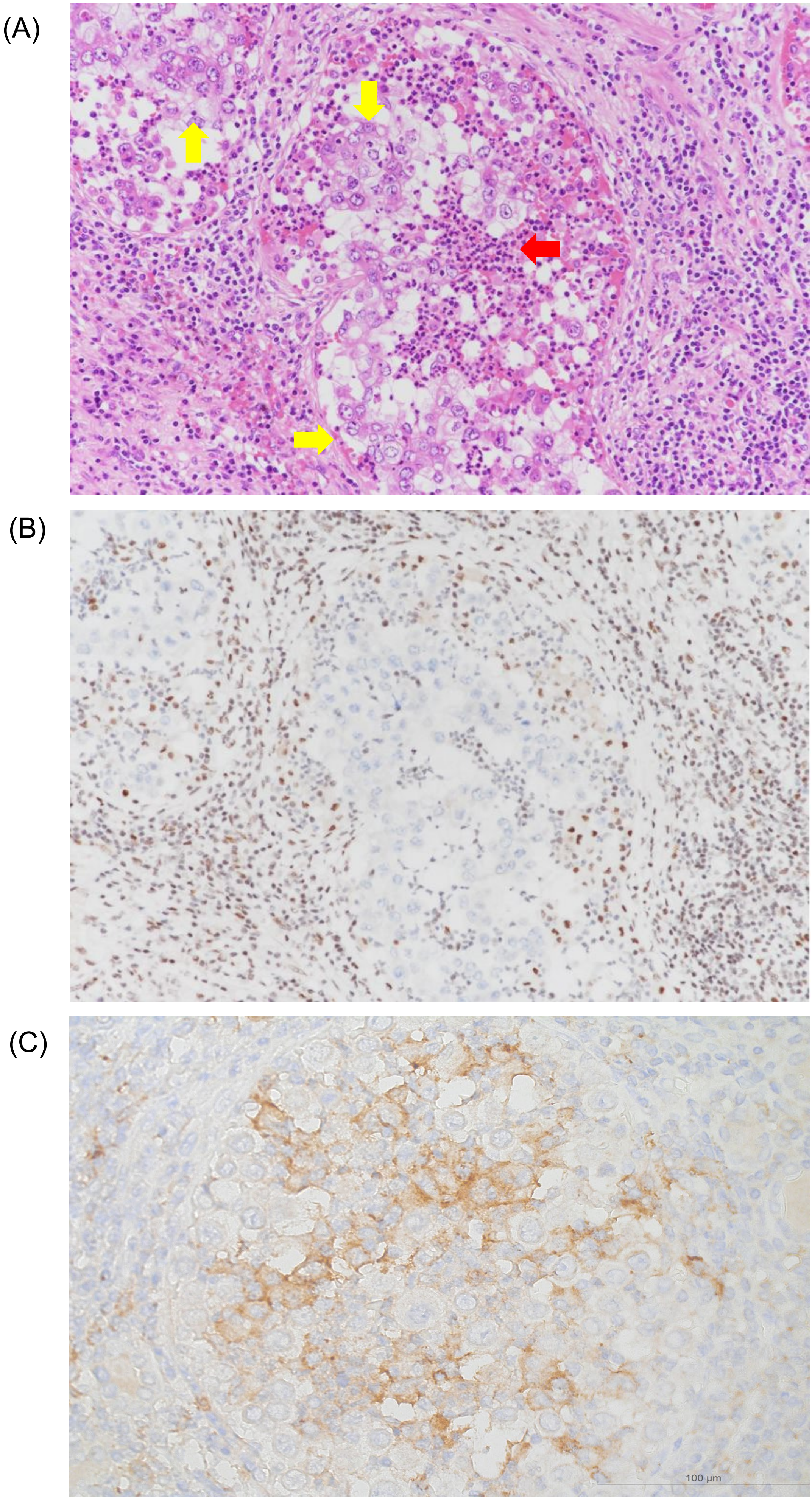

Supplementary data 2

Lymph node	Baseline (mm X mm)	Induction therapy (mm X mm)	Maintenance therapy (mm X mm)		After ICI (mm X mm)
			4 cycles	20 cycles	
Para aorta	41 × 34	31 × 29	31 × 22	21 × 16	24 × 14
supraclavicular	24 × 20	12 × 10	11 × 8	9 × 6	8 × 6
Retro crural	16 × 12	8 × 7	7 × 6	9 × 5	9 × 5

Supplementary data 3

(A) Shift in $CD8^+$ T cell subsets during ICI treatment

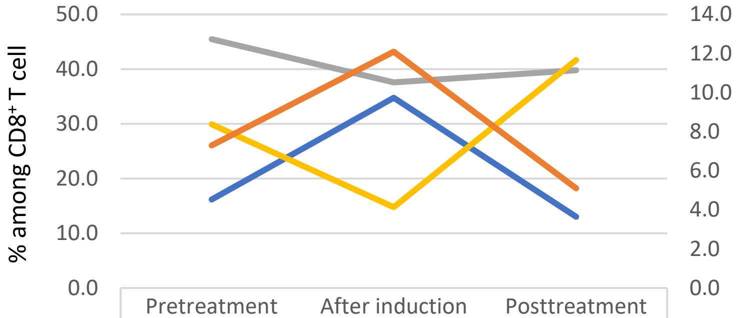

	Pretreatment (%)	After induction (%)	Posttreatment (%)
naïve	16.2	34.8	13.0
Tem	45.5	37.6	39.8
Teff	29.9	14.8	41.7
Tcm	7.3	12.1	5.1

(B) Change in CTLA-4 expression of $CD8^+$ T cell subsets during ICI treatment

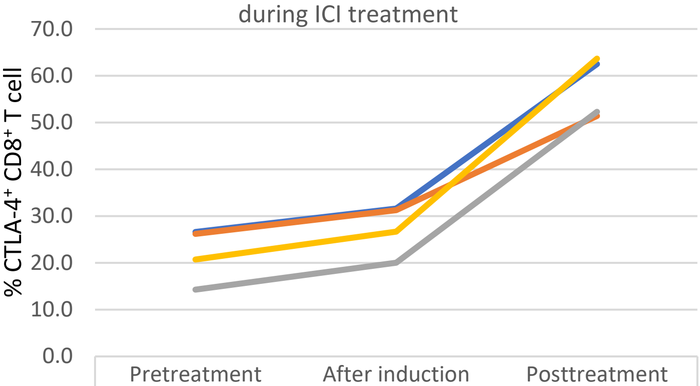

	Pretreatment (%)	After induction (%)	Posttreatment (%)
naïve	26.6	31.6	62.5
Tcm	26.2	31.3	51.4
Tem	14.3	20.0	52.3
Teff	20.7	26.7	63.7

Change in PD-1 expression of $CD8^+$ T cell subsets during ICI treatment

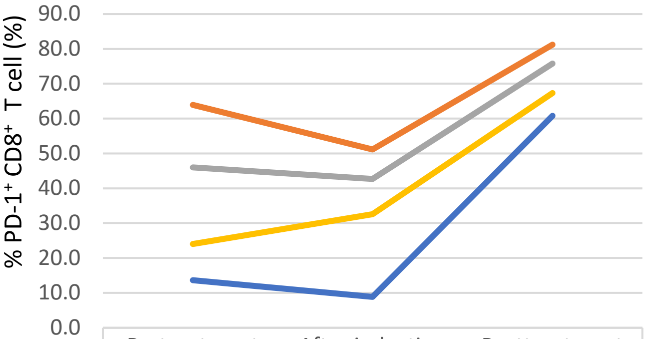

	Pretreatment (%)	After induction (%)	Posttreatment (%)
naïve	13.6	8.8	60.8
Tcm	63.9	51.1	81.2
Tem	46.0	42.7	75.8
Teff	24.0	32.6	67.3

Change in LAG-3 expression of $CD8^+$ T cell subsets during ICI treatment

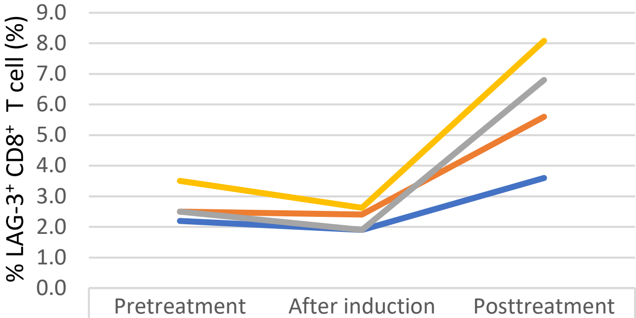

	Pretreatment (%)	After induction (%)	Posttreatment (%)
naïve	2.2	1.9	3.6
Tcm	2.5	2.4	5.6
Tem	2.5	1.9	6.8
Teff	3.5	2.6	8.1

Change in PD-1, CTLA-4, LAG-3 expression of Th1 cell during ICI treatment

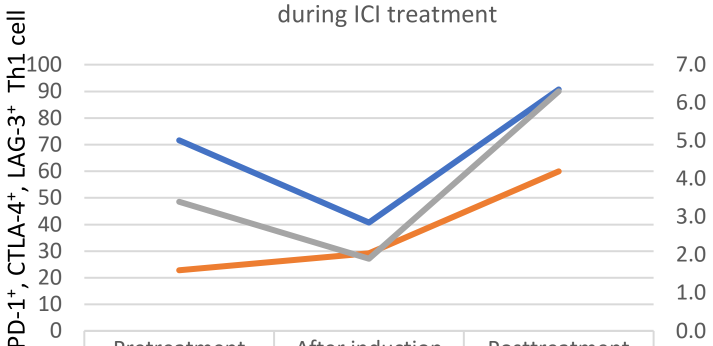

	Pretreatment (%)	After induction (%)	Posttreatment (%)
PD-1	71.6	40.7	90.7
CTLA-4	22.8	29.2	60
LAG-3	3.4	1.9	6.3

Supplementary data 4

Author	N	T stage	Metastatic site	treatment	outcome	Overall survival (months)
Lai et al.	1	T3	Lung	Everolimus, Avastin	N/A	N/A
Sirohi et al.	5	T3 5/5	Lymph node 4/5	N/A	DOD 4/5	3-27
Colombo et al.	1	T3	Lung, Lymph node	Sunitinib, Sorafenib	DOD	10
Our case	1	T3	Lymph node	Ipilimumab Nivolumab	DOD	28
